# Ovulatory status of overweight women without Polycystic Ovary Syndrome

**DOI:** 10.5935/1518-0557.20180071

**Published:** 2019

**Authors:** Reinaldo S. A. Sasaki, Mário S. Approbato, Mônica C. S. Maia, Eliamar A. B. Fleury e Ferreira, Neuma Zanluchi

**Affiliations:** 1 Assisted Reproduction Laboratory - Hospital das Clínicas - Federal University of Goiás; 2 Federal University of Goiás; 3 Reference Center for Radioactivity – SES- GO

**Keywords:** ovulation, ovulation detection, ultrasonography, overweight, obesity

## Abstract

**Objective:**

Obesity is one of the extra hypothalamic-pituitary-ovarian axis factors that can influence ovulation. The isolated impact of obesity on ovulation without other comorbidities needs to be further studied. Our goal is to evaluate the association between the anovulation in the ultrasonographic monitoring of the ovulation cycle and the body mass increase of infertile patients without polycystic ovaries of a university service.

**Methods:**

Case-control study performed at the Human Reproduction Laboratory of the University Hospital. We evaluated 1,356 ultrasound monitoring reports of ovulation between January 2011 and December 2015. We named case those patients who ovulated on the monitored cycle. After applying the exclusion criteria, we consolidated a total of 110 cases and 118 controls. The exposure variables were normal BMI or patients classified with a BMI above normal. Data analysis was performed using SPSS 22.0. Differences in proportions were assessed by X^2^ test Pearson, Fisher and Wilcoxon test. The value of *p*<0.05 was considered statistically significant.

**Results:**

The groups were comparable in age, age at menarche, number of pregnancies, deliveries, cesarean sections and abortions, number of antral follicles, FSH, prolactin and TSH values. Among the anovulatory patients, 57 (51.82%) were overweight, while among ovulatory patients, 44 (37.29%) were in this same BMI category. The odds ratio was 1.8655, with a significant p value (*p*<0.05).

**Conclusion:**

There was an association between anovulation and increase in the Body Mass Index, with an increased risk of anovulation in patients with BMI above normal.

## INTRODUCTION

Menstruation is a cyclic endometrial desquamation resulting from the interactions of hormones produced by the hypothalamic-pituitary-ovarian axis. The integrity of the hypothalamic-pituitary-ovarian axis is essential for ovulation and regular menstrual cycles, and external factors may influence this complex feedback system (Speroff & Fritz, 2005). Since the primary function of the menstrual cycle is to promote a woman's reproductive capacity, changes in this cycle impact on female fertility. Rogers & Mitchell (1952) demonstrated an association between menstrual changes, excess weight, infertility, and recurrent miscarriages. Therefore, studies on the association of menstrual irregularity and obesity are long-standing; however, many of these studies have been limited because of their small sample size and the participants have gynecological problems or are participating in weight reduction programs.

Obesity is one of the factors that can influence the menstrual cycle. It can be defined as a disease characterized by excessive accumulation of body fat, due positive energy balance, causing health effects, with significant loss in quality and lifetime. One of the classifications of obesity was proposed by the World Health Organization (WHO, 2015). Several pathophysiological disorders are caused by obesity, especially in people with Body Mass Indexes (BMI) above 30 kg/m^2^. Cardiovascular disorders, endocrine and metabolic disorders, respiratory disorders, gastrointestinal disorders, dermatological disorders, musculoskeletal disorders, neoplasia psychosocial disorders, increased surgical and anesthetic risk, decreased physical agility and disorders in pregnancy and fertility, as absence of ovulation (Tavares *et al*., 2010). In the United States, about 68% of the adult population is overweight, and about 34.9% are obese (Ogden *et al*., 2014). According to the Brazilian Ministry of Health, in the adult population in Brazilian capitals, overweight prevalence is of 49.1% among women. In Goiânia, the state capital of Goiás (Brazil), 48.1% of women are overweight and 16.3% are obese (Brazil-Ministério da Saúde, 2015).

Some studies have used self-reported height and weight; and the definitions of menstrual regularity vary according to each study. Much of this research is limited to overweight and obese patients associated with Polycystic Ovarian Syndrome (PCOS). Due to the increasing prevalence of overweight and obesity, it is important to investigate its effects on the reproductive health of women, and to better quantify the strength of associations with menstrual irregularity (Wei *et al*., 2009). The early onset of obesity, especially in adolescence, favors the development of irregular cycles, oligo/chronic anovulation and infertility in adults (Pasquali & Gambineri, 2006). In addition to the deleterious effects of obesity on female reproductive function, such as delay in spontaneous conception, higher prevalence of infertility and natural abortions, there is still a worse response to infertility treatments and a higher prevalence of obstetric complications (Pasquali & Gambineri, 2006; Nelson & Fleming, 2007).

The subject of this study was the ecograph assessment of ovulation and menstrual cycle parameters, associating them to higher BMI in infertile patients. The problem is the high prevalence of overweight and obese women in the world, in Brazil and also in Goiânia, and the repercussions on their health, especially in relation to their reproductive health. There is also an increase in childhood obesity, and there may be an increase in sub and infertility in the future. This study is justified, because although there are several worldwide studies on menstrual irregularity and obesity, there are few with hospital samples involving assisted reproductive clinics accompanying the cycle and ovulation of these patients through the monitoring of ovulation, and the great majority is patients with obesity and PCOS concomitantly.

Due to the increasing prevalence of overweight and obesity, it is important to investigate their effects on the reproductive health of women and quantify the strength of association with ovulation and menstrual irregularity (Wei *et al*., 2009). Since menstrual regularity is closely associated with endocrine physiology and ovulation, the assessment of the association of these factors with body mass index (BMI) will help on this quantification. Various researches invariably associate obesity with polycystic ovaries thus creating a bias. We reinforce that patients with polycystic ovaries are not included; demonstrating that obesity alone is an association factor to anovulation. The objective of this study is to assess whether there is an association between the presence or absence of ovulation and the presence of overweight/obesity calculated by BMI in infertile patients without Polycystic Ovary Syndrome, in a university service.

## MATERIALS AND METHODS

This was a case-control study performed at the Human Reproduction Laboratory of the University Hospital (Lab Rep HC/UFG). We evaluated 1,356 monitoring of basal ovulation, without stimulation with ovulation inducers, performed between January 2011 and December 2015. For "n" calculation, with 80% power test, the number of cases and controls needed was 105 each.

After collecting the data from the ovulation monitoring form, we selected the cases as the anovulatory patients in the cycle. The study included patients with infertility for at least one year, aged 18 to 38 years, no more than 10 mm follicle on 1^st^ examination, antral follicle count between 3 and 12 in each ovary counted between the 2^nd^ and the 5^th^ day of the cycle, absence of prior cystectomy or oophorectomy, and endometriomas absence. We selected 148 cases.

The control group, consisting of reports of patients who ovulate on the monitored cycle, was randomly selected, and we applied the same inclusion criteria used for the group of cases. Ovulation in the control group was considered only in those cycles in which the follicle reached at least 16 mm in diameter. The disappearance or decrease of at least 70% of the diameter was considered follicular collapse. We selected 154 controls.

Later, we assessed the medical records of patients. The inclusion and exclusion criteria were based on the fact that they are factors that influence the menstrual cycle and fertility of women (confounders). After applying the exclusion criteria, we ended up having 110 cases (patients who did not ovulate on the monitored cycle), and 118 controls (patients who ovulated on the monitored cycle). The exclusion criteria were patients with low body weight (BMI <18.5 kg/m^2^); follicle stimulating hormone (FSH) above 10IU/L; stages III and IV endometriosis diagnosis, Thyroid Stimulant Hormone (TSH) lower than 0.4 or higher than 4.5mU/L; prolactinaemia above 20 ng/ml; diagnostic Polycystic Ovary Syndrome and smokers. At this point we had access to BMI data of patients, checking for the presence or absence of exposure factors (overweight/obesity). Henceforth, the presence of overweight or obese patients will be considered "BMI above normal".

**Figure 1:**
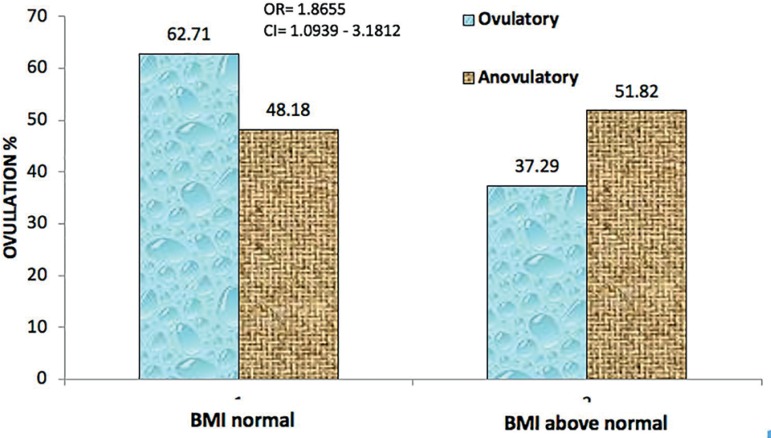
Flow chart assessing the presence of ovulation in overweight and normal individuals. Lab Rep HC-UFG 2016

The ovulatory status of the study variables was: absence or presence of a monitored ovulation in the cycle. The exposure variables were: normal BMI or patients classified with a BMI above normal (Figure 1).

The patients were classified according to BMI, following the WHO’s definition of body mass (18.5 to 24 kg/m^2^), and above normal (>25 kg/m^2^) (WHO, 2015).

The patients were subjected to biometric examination, assessing height and weight on a WELMY, W110H model scale. Ovulation monitoring was performed on the 2^nd^ to the 5^th^ day of the cycle, with new measurements from the 10^th^ day of the cycle until ovulation occurs, or until day 16 if there is no dominant developing follicle(s) or follicular collapse. Antral follicles were counted following the technical recommendations of Broekmans *et al*. (2010) between the 2^nd^ and the 4^th^ day of the spontaneous menstrual cycle. The ultrasound equipment used was the LOGIQ P6 model, manufactured by General Electrics (GE). The examinations were performed by physicians from the Human Reproduction Laboratory HC-UFG.

Comparability between the case and control groups was confirmed by checking the pairing of the following variables: age (years); age at first menstruation (menarche); number of pregnancies; number of children born up to 22 weeks of gestational age (Parity); number of abortions; the number of antral follicles between the 2^nd^ and 5^th^ day of the cycle; having bilateral tubal ligation (BTL)and the laboratory test values of FSH (IU/L), prolactin (mcg/L) and TSH (mU/L).

In order to compare the mean values between the case and control groups, because they were two unrelated groups, we chose the t-test for independent samples as a parametric test for the variables with normal distribution and the Mann-Whitney test as a non-parametric test for variables with non-normal distribution (Mann & Whitney, 1947).

To assess whether the patients in the case group and the control group had a different BMI on the WHO classification between normal weight or overweight, we used the Pearson’s performed chi-square statistical test (χ^2^) and calculated the Odds Ratio. For the statistical analysis we used the SPSS 22.0 software.

In the study, we used the database of medical records of patients from the Human Reproduction Laboratory of the Clinica's Hospital, Federal University of Goiás. No informed consent of patients was needed. The study was approved by the Ethics Committee of the Clinica's Hospital, Federal University of Goiás under number 1,235,590.

## RESULTS

After assessing the statistical tests of case and control groups to see if they were comparable, we found out that the variables matched, so the groups were paired; thus, there was no need for adjustments (Table 1).

**Table 1:** Characteristics of the two study groups. Lab Rep HC/UFG 2016

	Case (n=110)	Control (n=118)	*P Value*
Age (years)	32.45±4.13	32.66±3.95	0.745*
Menarche (years)	12.56±1.75	12.61±1.67	0.621*
Pregnancies	1.25±1.50	1.36±1.36	0.346*
Parity	0.95±1.22	0.99±1.26	0.844*
Abortions	0.29±0.76	0.37±0.74	0.191*
Number of antral follicles	12.86±4.37	13.32±3.86	0.294*
FSH (UI/l)	6.11±1.98	5.92±1.61	0.425**
Prolactin (mcg/L)	12.17±4.04	12.09±3.98	0.957*
TSH (mU/L)	1.82±0.749	1.775±0.844	0.350*

Lab Rep HC/UFG= Human Reproduction Laboratory of the Clinica's Hospital; n=number;

* Mann-Whitney test;

** t-Test

The height of anovulatory patients ranged between 1.40 and 1.80 m (x=1.60±0.07), the weight between 41.5 and 105kg (x=65.60Kg±12.52) and BMI ranged between 18.11 and 38.96kg/m^2^ (x=25.64±4.24Kg/m^2^). In the group of ovulatory patients, height was between 1.45 to 1.73m (x=1.60±0.063), weight between 45.2 and 97.5Kg (x=63,29Kg±10.77) and BMI between 18.25 and 36.40 kg/m^2^ (x=24,76Kg/m^2^±3.81).

**Figure 2:**
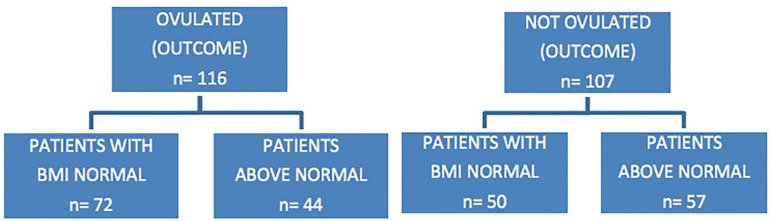
Distribution of 228 patients according to ovulation categorized by BMI. Lab Rep HC/UFG 2016

Table 2 and Figure 2 depict the distribution of patients, according to ovulation, categorized by BMI. From a total of 228 patients monitored, 110 were anovulatory and 118 were ovulatory. Among the anovulatory patients, 57 (51.82%) were overweight; while among ovulatory patients, 44 (37.29%) were in this same BMI category. The odds ratio was 1.8087, with a significant *p* value (*p*<0.05).

**Table 2:** Distribution of 228 patients according to ovulation, categorized according to BMI. Lab Rep HC/UFG 2016.

	n	Anovulatory (case)	Ovulatory (control)	*OR*	CI	p* Value
BMI above normal	101	57	44	1.8087	1.0664 – 3.0677	0.0273
BMI Normal	127	53	74			
Total	228	110	118			

Lab Rep HC/UFG= Human Reproduction Laboratory of the University Hospital; n= number;

* *X*^2^= 4.871, Pearson’s test; OR= *Odds Ratio*; CI= Confidence Interval.

## DISCUSSION

Infertility is a disease of the reproductive system, defined by the inability to achieve clinical pregnancy after 12 months or more of regular and unprotected sexual intercourse (ASRM, 2013). WHO estimates that 48.5 million couples worldwide are infertile. Ovulatory infertility can reach up to a quarter of infertility etiologies. Since obesity affects ovarian function, evaluating body mass parameters are very relevant for infertile patients (Mascarenhas *et al*., 2012; Nardo & Chouliaras, 2015). Ovulation sets the cycle regularity, and both int raovarian factors regulate folliculogenesis, and there should be a balance between them. Any imbalance between the extra and intra ovarian factors may result in abnormal folliculogenesis (NICE, 2013). However, the clinical evaluation of menstrual regularity, may not be a reliable parameter for the diagnosis of ovulation. The assessment of ovulation by ultrasound provides for a more accurate diagnosis of ovulation (Frank *et al*., 2008).

Obesity is a complex and multifactorial disease, developed by the interaction between the person's genotype and the environment (Malcolm & Cumming, 2003; NHI, 2000). The most obese women do not have fertility disorders, but obesity may negatively influence their menstrual cycle and fertility; and one mechanism, is the absence of ovulation (ASRM, 2008). Beyond its risks during pregnancy, obesity on puerperium is associated with the occurrence of infectious complications in the postpartum period, such as infection of surgical wounds, urinary tract infection and need for antibiotics (Chin *et al*., 2014).

The study was carefully concocted, and the selection bias was mitigated by the strictness of the inclusion and exclusion criteria. Group comparability was ensured by the assessment of pairing the possible confounding variables. The observer bias was mitigated because there was blinding about the exposure factor in both the study group and the control group, avoiding bias in information collection. Although most obese women do not develop infertility, when obesity influences cycle control and ovulation, it can have a negative impact on female fertility (Paiva *et al.*, 2012). With the increasing prevalence of obese and overweight children and women, the interference of those with menstrual regularity, and consequently on fertility, acquires greater importance. There are many studies involving infertile patients with overweight and obesity, but the samples in most cases include or are limited to patients with polycystic ovaries. In this study, the lack or absence of ovulation was assessed by analyzing the body mass index of patients, excluding those with polycystic ovaries.

Our results were coincident with many studies in the literature, such as Kuchenbecker *et al*. (2010), who reported an association between anovulation and overweight. It also corroborated by other studies that indicate an increased risk of oligo and anovulation in obese women, such as those from Brewer & Balen (2010) and Oliveira & Lemos (2010), even among those women who have regular menstrual cycles. Obesity was also associated with oligomenorrhea and anovulation by Yilmaz *et al*. (2009). The chance of patients with higher-than-normal BMI not ovulating is about 1.8 times greater than among normal BMI individuals.

It is possible that both the excess weight and infertility be symptoms of the same pathology. Although obesity decreases fertility, it is unclear how much weight loss could increase it in overweight patients (Koning *et al.,* 2010). The fertility treatment in subfertile women with overweight and obesity differs between countries and treatment centers. In some centers in the Netherlands, overweight women are not treated at all. At other fertility centers, overweight and obese women are treated regardless of their BMI. The British Fertility Society inform women with a BMI above 30 kg/m^2^ that they tend to take longer to get pregnant, and if they are not ovulating weight loss increases the chances of conception. While there is enough convincing evidence that weight reduction eventually leads to more spontaneous pregnancies, the main goal is to reduce the complications of excess weight during pregnancy (NICE, 2013).

The American Society for Reproductive Medicine and the American College of Obstetrics did not publish guidelines for clinical management of obese infertile patients (Vahratian & Smith, 2009). The institutions that restrict the treatment of overweight patients justify the restrictions because of high costs and limited funding for the procedures, decreasing success and increasing risk of complications during pregnancy (Koning *et al.,* 2010). Restrictions on fertility treatments in patients with high BMI often do not address issues of justice and may violate a woman's right to autonomy. Those patients with increased BMI can be discriminated and without access to treatment.

Our study indicates that patients with excess body mass, without other diseases, were associated with anovulation. This is a contribution to clarify these issues. We considered as a limiting factor, the fact that in our study, monitoring of ovulation is performed in a single cycle, and there may be variations between the follicular growth cycles in the same woman. However, monitoring during one cycle only is the procedure adopted by most human reproduction services (Mikolajczyk *et al*., 2008).

## CONCLUSIONS

There was an association between the lack of ovulation upon ultrasound monitoring of the ovulation cycle and increased body mass index, with higher anovulation risk in patients above normal weight, even if they did not have polycystic ovaries.
